# Draft Genome Sequence of *Cronobacter sakazakii* GP1999, Sequence Type 145, an Epiphytic Isolate Obtained from the Tomato’s Rhizoplane/Rhizosphere Continuum

**DOI:** 10.1128/genomeA.00723-17

**Published:** 2017-08-03

**Authors:** Hannah R. Chase, Leo Eberl, Roger Stephan, HyeJin Jeong, Chaeyoon Lee, Samantha Finkelstein, Flavia Negrete, Jayanthi Gangiredla, Isha Patel, Ben D. Tall, Gopal R. Gopinath, Angelika Lehner

**Affiliations:** a Center for Food Safety and Applied Nutrition, U.S. Food and Drug Administration, Laurel, Maryland, USA; b Department of Plant and Microbial Biology, University of Zurich, Zurich, Switzerland; c Institute for Food Safety and Hygiene, University of Zurich, Zurich, Switzerland

## Abstract

We present here the draft genome of *Cronobacter sakazakii* GP1999, a sequence type 145 strain isolated from the rhizosphere of tomato plants. Assembly and annotation of the genome resulted in a genome of 4,504,670 bp in size, with 4,148 coding sequences, and a GC content of 56.8%.

## GENOME ANNOUNCEMENT

*Cronobacter* spp. are food-associated pathogens that cause rare but severe cases of meningitis, necrotizing enterocolitis, sepsis, and pneumonia in preterm and/or immunocompromised infants (1–3). The genus comprises seven species—*C. sakazakii*, *C. malonaticus*, *C. turicensis*, *C. universalis*, *C. condimenti*, *C. muytjensii*, and *C. dublinensis*—all capable of infecting humans, with the exception of *C. condimenti* (4, 5). *Cronobacter* spp. have been isolated from a variety of environmental sources like soil, household dust, and powdered infant formula production lines, as well as from fruits, vegetables, herbs, cereals, and grains (6–8), and they have also been isolated from lemon tree, wheat, rice, and soybean plant rhizoplane/rhizosphere continuums (9–12).

Several lines of evidence suggest environmental origins for *Cronobacter* spp., with plants as the ancestral econiche promoting the diversification of this genus (13, 14). In this report, we present the genome sequence of *C. sakazakii* GP1999—originally isolated in 1999 from the roots of a *Lycopersicon esculentum* (tomato) plant by Schmid et al. (13).

GP1999 genomic DNA was subjected to whole-genome sequencing using the MiSeq platform (Illumina, San Diego, CA, USA) and a Nextera XT library kit. *De novo* assembly with CLC Genomics Workbench version 7.0 (CLC bio, Aarhus, Denmark) resulted in a genome of 4,504,670 bp, with 22 contigs and a GC content of 56.8%. The genome was annotated using the RAST annotation server, and 4,148 coding sequences were identified (15, 16). The *Cronobacter* sp. multilocus sequence typing website (http://pubmlst.org/cronobacter) showed that *C. sakazakii* GP1999 belonged to sequence type 145 (17).

The strain harbors a pESA3/pSP291-like plasmid, which was found by comparing its genome assembly with whole-genome sequences of strains *C. sakazakii* BAA-894 (NC_00978) and *C. turicensis* z3032 (NC_01328) and confirmed by PCR analysis. However, pESA2-like and pCTU-3 plasmid replicons were not detected by PCR (18).

Other mobilome genes found in the assembly include a total of 11 integrase/transposase genes, 8 genes encoding unspecified mobile element proteins, and 63 genes encoding phage-associated proteins. Notably, a gene encoding resistance to the antibiotic fosfomycin was found downstream of a transposase.

Other genes identified in GP1999 include virulence-associated genes encoding the protein MsgA and factors VirL and MviM (15, 16), as well as multidrug resistance efflux pump-related genes belonging to the *acr*AB operon, the resistance-nodulation-division family, the major facilitator superfamily, and tripartite systems. Additionally, genes encoding heavy metal resistance to copper, organic hydroperoxide, fusaric acid, and tellurite were found. Interestingly, an arsenic resistance operon repressor gene was identified downstream to an arsenic efflux pump operon and an arsenate reductase. An albicidin (a phytotoxin that blocks DNA gyrase in chloroplasts) resistance protein (19) was also observed. Furthermore, strain GP1999 contains a 16,771-bp operon encoding a xylose utilization pathway, supporting the hypothesis that plants represent the ancestral econiche of *Cronobacter* spp.

To the best of our knowledge, this is the first genome of a plant isolate of *C. sakazakii* being reported. The availability of the GP1999 genome will enable comparison with other genomes of *C. sakazakii* strains, thereby providing better insights into the genetic features linked to plant association and possibly the natural history of this important foodborne pathogen.

### Accession number(s).

The whole-genome sequence of *C. sakazakii* GP1999 has been submitted to NCBI GenBank under the accession number NHTW00000000 (*Cronobacter* GenomeTrakr FDA-CFSAN BioProject number PRJNA258403). The version described in this paper is the first version, NHTW01000000.
